# Aerosol Survival, Disinfection and Formalin Inactivation of Nipah Virus

**DOI:** 10.3390/v14092057

**Published:** 2022-09-16

**Authors:** Sophie J. Smither, Lin S. Eastaugh, Lyn M. O’Brien, Amanda L. Phelps, Mark S. Lever

**Affiliations:** Chemical Biological and Radiological (CBR) Division, Defence Science and Technology Laboratory (DSTL), Porton Down, Wiltshire SP4 0JQ, UK

**Keywords:** Nipah virus, emerging viruses, disinfectant, inactivation, aerosol

## Abstract

Nipah virus is a relatively newly discovered emerging virus on the WHO list of priority pathogens which has the potential to cause outbreaks with high fatality rates. Whilst progress is being made in the development of animal models for evaluating vaccines and therapies, some of the more fundamental data on Nipah virus are lacking. We performed studies to generate novel information on the aerosol survival of Nipah virus and to look at the efficacy of two common disinfectants. We also performed studies to evaluate the inactivation of Nipah virus by using neutral buffered formalin. Nipah virus was relatively stable in a small particle (1–5 µm) aerosol in the dark, with it having a decay rate of 1.46%min^−1^. Sodium hypochlorite (at 10%) and ethanol (at 80%) reduced the titre of Nipah virus to undetectable levels. Nipah virus that was in tissue culture medium was also inactivated after 24 h in the presence of 10% formalin.

## 1. Introduction

Nipah virus (NiV) is a zoonotic RNA virus of the Paramyxoviridae family and the Henipavirus genus [1]. A relatively new, emerging pathogen which was discovered at the turn of the 20th century, NiV is found in South-East Asia and India and causes a respiratory and encephalitic disease which has high case-fatality rates. The reservoir for NiV is through fruit bats, and the original outbreak of NiV in Malaysia was associated with contact with pigs (who likely consumed contaminated fruits that were left by bats) [2]. More recent outbreaks have been associated with date palm fruit harvesting or consumption, as well as contact with bats and their secretions [2]. The human-to-human transmission of NiV has been reported through close or direct contact with infected individuals or their secretions, including respiratory secretions [2].

NiV is on the WHO High Priority pathogens list which means it is amongst those “diseases that pose the greatest public health risk due to their epidemic potential and/or where there is no or insufficient countermeasure”, and it is therefore in need of urgent Research and Development action [3]. As the pathogen has high case-fatality rates and can spread from person-to-person, there is also the potential that it could be used as a biological weapon, which is considered in [4]. Whilst significant progress has been made in the development of relevant animal models for testing vaccines and antiviral treatments, there is less information on other, more fundamental aspects of the virus that may be important in outbreak situations, or for people who are working with the virus. NiV must be handled in BSL-4 containment laboratories which means that there are only a limited number of laboratories world-wide that are capable of working with the wild-type virus. As NiV can spread from person-to-person and it can be found in respiratory secretions, data on the survival characteristics of NiV in aerosols are needed to contribute to the understanding of the potential role of the aerosolised virus in transmission, and to understand the hazard that is posed by the aerosolised virus that may be generated deliberately [4], or during laboratory or medical practices [5,6]. There are minimal amounts of published data on the disinfection of NiV, although disinfection is a vital control measure in outbreak settings. Similarly, there are limited numbers of published data on the chemical inactivation of NiV, which are useful for those working with the virus. 

In this paper, we present novel data on the survival of NiV in small particle aerosols and demonstrate the efficacy of bleach and alcohol-based disinfection through the testing of reduction in NiV titre after short contact times with a range of concentrations of sodium hypochlorite and ethanol. We also present our data on the inactivation of NiV by neutral buffered formalin which has enabled us to maximise data from in vivo studies by allowing the safe handling of formalin-fixed tissues from animals that are infected with NiV.

## 2. Materials and Methods

### 2.1. Virus Growth and Enumeration

Nipah virus-Malaysia (hereafter referred to as NiV) was kindly provided by Viral Special Pathogens Branch, (CDC) where it was derived from the “199901924” isolate and had undergone 2 passages in Vero-E6 cells and one passage in RK13 cells. At Dstl, the virus was passaged a further two times in Vero C1008 cells (ECCAC Cat. No. 85020206), and it was maintained in the tissue culture media (TCM) to produce a working stock of 2 × 10^6^ TCID_50_/mL. The TCM was Dulbecco’s minimum essential media (Sigma, Gillingham, UK) which was supplemented with 2% Fetal Calf Serum, 1% L-glutamine and 1% Penicillin/streptomycin (all Sigma). 

NiV was titrated by a 50% tissue culture infectious dose (TCID_50_) assay to enumerate a viable virus as described previously [7]. Briefly, the virus was added to the first column of a 96-well cell culture plate of confluent Vero C1008 cells. Serial ten-fold dilutions were made across the plate. The plates were incubated at 37 °C and 5% CO_2_ for 5–7 days and observed under a microscope for the presence of cytopathic effects (CPE). Each column of 8 wells of cells was scored for the presence or absence of CPE and the 50% end-point was calculated by the methods of Reed and Muench [8].

For the passaging of samples, flasks of increasing size were used for sequential passage. All of the flasks (Corning, Flintshire, UK) had a confluent monolayer of Vero C1008 cells which was maintained in TCM that was supplemented with 2% Fetal Calf Serum, 1% L-glutamine and 1% Penicillin/streptomycin. The final volumes in the flasks were 5 mL (T12.5 flasks), 10 mL (T25 flasks), 20 mL (T75 flasks) or 30 mL (T150 flasks). The flasks were incubated at 37 °C and 5% CO_2_ for 5–7 days for each passage. In order to establish the limit of detection of viable NiV in the flasks, a ten-fold dilution series was prepared in 1 mL media and the entire range of the dilution was added to individual flasks, and each flask was passaged three times alongside the test samples. The microscope that used to visualise cells and look for CPE was the Leica DMIRB. Microscope photos were taken using the Leica DFC310FX camera and captured using Leica Application Suite software v4.9 (Leica Microsystems (UK) Ltd., Milton Keynes, UK). 

### 2.2. Aerosol Survival in the Goldberg Drum

Studies to determine virus survival in small particle aerosols were performed using the rotating 40 L Goldberg drum as described previously [9,10,11], within an Advisory Committee on Dangerous Pathogens (ACDP) Containment Level 4 (equivalent to BSL4) laboratory. Briefly, the AeroMP (BiAera Technologies, LLC, Maryland, US) and a 3 jet Collison nebuliser (known to produce particles in the range of 1–5 µm) [12] were used to generate and condition an aerosol at 30 L/min and 50% relative humidity. The aerosol conditions in the drum was set to 50% relative humidity and 20 °C, and it was kept in the dark. Impinger sampling was performed at 4 L/min for 1 min using midget impingers, and these were collected into 3 mL TCM. A volume of 8 mL NiV stock was sprayed for 5 min to fill the drum, and the contents were then mixed for 2 min prior to the first (“T0”) sample being taken. The impingers were collected at various points over 90 min. The sprays were performed on three separate occasions, and at each time-point, the impingers were assayed by a TCID_50_ assay in triplicate, making a total of 9 values for each sample point. All drum experiments were performed in the dark, at an ambient temperature (19–21 °C) and a relative humidity (42–57%). In separate validation experiments, *Bacillus atrophaeus* (BA) spores were aerosolised under the same conditions as the virus was, as is described above. The BA spores at 10^8^ cfu/mL were diluted 1:10 in Phosphate Buffered Saline (PBS, Gibco, Thermo Fisher, Loughborough, UK) prior to aerosolisation. The impinger samples were collected in a PBS, and viable counts were performed on L-agar plates. For the aerosol data analysis, each time point value was corrected for the dilution effect of removing the air. The data was log_10_ transformed, and a best-fit line was determined using GraphPad Prism v 8 (GraphPad software, San Diego, CA, USA). 

### 2.3. Formalin Inactivation Testing

Ultra-centrifugation was used to remove formalin from the samples. For each round, 1 mL 20% formalin (Sigma) was added to 1 mL NiV virus stock, in triplicate (resulting in a 10% formalin/ 4% final concentration of formaldehyde). Two positive control samples were made by adding 1 mL TCM to 1 mL NiV virus stock, and the final sample was a negative control of 1 mL 20% formalin + 1 mL TCM. All samples were incubated at room temperature for 24 h. After 24 h, 30 mL TCM was added to each aliquot in ultracentrifuge tubes, then, 3 mL 30% sucrose was dispensed beneath the media to form a sucrose cushion. All of the samples were ultra-centrifuged at 25,000 rpm for 3.0–3.5 h at 4 °C (Beckman Optima Ultracentrifuge with SW28 rotor). After centrifugation, the supernatant was discarded, and a second wash was performed in the same manner as described above. The final pellets of the formalin-treated samples were re-suspended in 0.4 mL TCM. The entire sample (100%) was added to a T75 flask of confluent Vero C1008 cells in 20 mL media. The flasks were incubated at 37 °C for 6–7 days and then the entire contents of each flask were passaged onto fresh Vero C1008 cells in a T150 flask in 20 mL fresh media (resulting in 40 mL total). The T150 flasks were incubated at 37 °C for 6–7 days and then the cell monolayers were observed using a light microscope, and the presence or absence of cytopathic effects were recorded by two individuals each time. For the production of positive control samples, the pellet, after two rounds of ultra-centrifugation, was resuspended in 0.5 mL TCM. A TCID_50_ assay was performed on a 1:10 dilution (0.1 mL) of the pellet, and the remaining 0.5 mL was inoculated into flasks and processed as above. The entire inactivation process was repeated on three further occasions. 

### 2.4. Disinfectant Testing

Sodium hypochlorite (supplied as 14% Cl_2_ in aqueous solution) (VWR, Lutterworth, UK) and ethanol (supplied as 96%, VWR) were prepared at a range of percentage amounts (all *v*/*v*) in tap water, based on the undiluted, supplied reagent being equal to 100%. Disinfectants were prepared at 2× concentration so that when they were mixed at a 1:1 ratio with the virus, they were at concentrations of 40%, 30% or 20% ethanol (*v*/*v*) or 10%, 1%, 0.5% and 0.1% (*v*/*v*) sodium hypochlorite, respectively. 

To test the virucidal activity of the disinfectants, 50 µL of the disinfectant (either ethanol or sodium hypochlorite) was added to 50 µL NiV stock (at 2 × 10^6^ TCID_50_/mL) in a polystyrene 24-well culture plate (Corning Costar, Flintshire, UK). For the production of positive controls and to determine the maximum amount of virus recovery, 50 µL TCM was added to 50 µL NiV stock. The samples were tested in at least duplicate on two or three separate occasions. After 1 min or 5 min, 1 mL TCM was added to the virus + disinfectant, and then the samples were centrifuged using a microcentrifuge at 10,000 rpm for 5 min. After centrifugation, the liquid was discarded and 1 mL fresh TCM was added to the tube. This was repeated twice. After the final centrifugation, 1 mL TCM was added to the tube and a TCID_50_ assay was performed on each sample. The centrifugation steps were required to dilute the chemical components that would otherwise cause toxicity in the cell-culture based enumeration assay, and this method is similar to the methods that have been described previously [13]. 

After the TCID_50_ assay was performed, the remainder of each replicate sample was pooled and inoculated into confluent T12.5 flasks which were passaged twice into T25 flasks, then T75 flasks as described previously [13]. In a parallel test, 200 µL un-diluted ethanol was added to 50 µL NiV for 1 min and processed as above to test ethanol at a final concentration of 80%.

### 2.5. Flask Limit of Detection Testing

The limit of detection of NiV in the cell culture was determined by performing a ten-fold dilution series of NiV in 1 mL TCM, and then inoculating the T12.5 flasks with the entire sample. The flasks were incubated and passaged as described above, and the final flasks observed for the dilution at which NiV no longer caused infection in the flasks.

## 3. Results

### 3.1. Survival of NiV in Small Particle Aerosols in the Goldberg Drum

**Figure 1:**
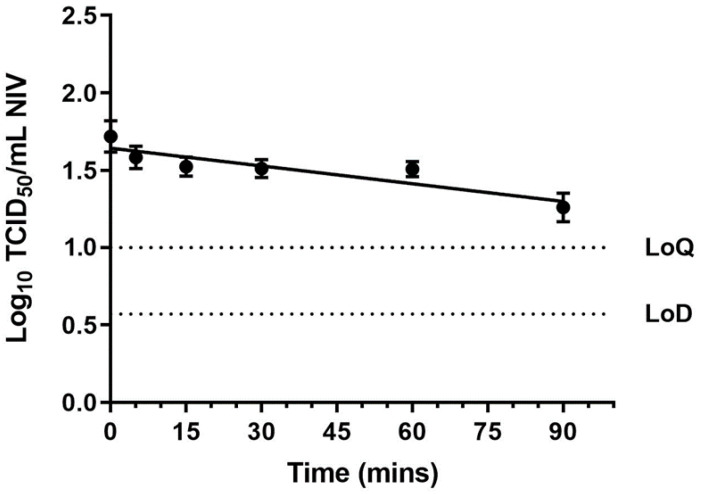
Survival of NiV in a small particle aerosol over time.

Small particle aerosols containing NiV were generated and held in the dark in a Goldberg drum over 90 min at ambient temperatures and a relative humidity. Impinger samples were collected at the following time intervals: 0, 5, 15, 30, 60 and 90 min, and were enumerated by a TCID_50_ assay in triplicate. The drum sprays were performed on three separate occasions. All drum counts were adjusted for the dilution effect and then log_10_ transformed (Figure 1). Non-linear regression was performed to determine the best fit line for the decay. The data suggested that NiV survived well within a small particle aerosol under the experimental conditions that have been described. Although the starting titres after aerosolisation were relatively low, viable virus was still detected in the aerosols at 90 min in all of the samples. The decay constant, K, for NiV was determined as −0.01475, which equated to a total decay rate of approximately 1.46 %min^−1^ (95% confidence intervals for k = −0.03236 to −0.006508). Based on this decay rate, the half-life of NiV in the drum is 47 min (95% CI for half-life = 21 to 107 min). Under these experimental conditions it would take 2.5 h (153 min) for the starting titre of virus to drop by 90% (a factor of 10).

NiV was aerosolised in TCM and held in a Goldberg Drum at a medium relative humidity (50–60%). Impinger samples were taken at set time points and enumerated by TCID_50_ assay in triplicate. Three separate aerosol experiments were performed. Counts were adjusted for the dilution effect of sampling. The mean adjusted count of virus ± SEM and a best fit line from all of the runs are plotted. The limit of detection (LoD) and the limit of quantification (LoQ) of the TCID_50_ assays are shown as dotted lines.

The validation studies using BA spores in the drum system that was described above indicated that there was minimal physical decay in the system (k = −0.003049, approximately 0.3 %min^−1^, 95% confidence intervals for k = −0.02672 to 0.007809). Taking into account the physical decay in the drum, the biological decay rate of NiV would be 1.16 %min^−1.^. This would then translate to a half-life of 60 min and it would take 198 min (3.3 h) for the titre to drop by 90%. 

### 3.2. Limit of Detection of NiV in Flasks

NiV was diluted 1:10 down to a −8 dilution, and all of the dilutions were used to infect T12.5 flasks. The flasks were passaged three times. The flasks that were infected with a −6 dilution of NiV stock (approx. 2 TCID_50_) reliably caused infection, whereas the flasks that were infected with a −7 dilution of NiV (approx. 0.2 TCID_50_) did not cause infection. This suggests that the LoD in the flasks is very low, at <2 TCID_50_ (results not shown). The limit of quantification of the TCID_50_ assay is 10 TCID_50_ (a result of 50% of the wells in the column containing a neat sample being infected). The limit of detection of the TCID_50_ assay is extrapolated to 3.73 TCID_50_ (based on a single well that was positive for infection in the column containing neat samples), therefore, the flasks are likely to be more sensitive in detecting a viable virus than the assay.

### 3.3. Inactivation of NiV by Neutral Buffered Formalin

NiV and neutral buffered formalin were mixed together for 24 h at room temperature and then the entire sample was washed (to remove toxicity to the cell culture), and then they were tested for viral inactivation by infecting the cells with 100% of the sample which was followed by a subsequent passage. The starting titre of NiV was 2 × 10^6^ TCID_50_/mL. The mean titre after one wash was 7.82 × 10^5^ TCID_50_/mL, and after two washes, this was 1 × 10^5^ TCID_50_/mL. Although there was a ten-fold reduction in the titre after washing, this was necessary to remove the formalin whilst retaining a sufficient amount of the virus to demonstrate viral inactivation. 

**Figure 2:**
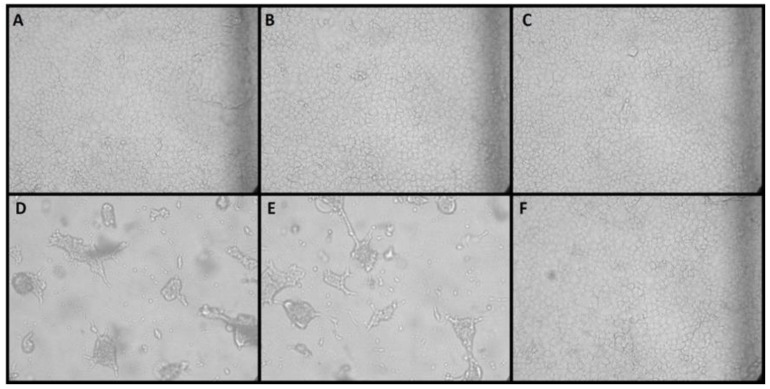
Inactivation of NiV by Formalin. NiV was incubated with either 10% neutral buffered formalin (**A**–**C**) or TCM (**D**,**E**) and 10% formalin was incubated with TCM (**F**). All incubation was for 24 h. After incubation, the samples were washed twice by ultracentrifugation and then added to T75 tissue culture flasks. Images show the cell monolayer after passage into T150 flasks. Infection (as seen in **D**,**E**) indicated that the virus was not inactivated. Intact monolayers (**A**–**C**) suggest that all viable virus has been inactivated.

NiV was inactivated by 10% formalin (4% formaldehyde) after 24 h incubation at room temperature. This method was reproducible; a total of 12 replicates were tested (three each time on four occasions). An example of the final cell monolayers is shown in Figure 2. NiV caused very obvious signs of infection in Vero C1008 cells after 3–4 days. Incubation for 6 or 7 days which was followed by passage gave low levels of the virus plenty of time to recover and cause cytopathic effects in the cell culture, but no signs of infection were observed in any of the flasks that were inoculated with the formalin-treated samples after 2 rounds of passage. The cells that were infected with NiV sham inactivated with TCM instead of formalin (positive control), and also washed twice in an identical fashion to the test samples always resulted in cytopathic effects being observed after the first passage. The cells that were infected with a TCM + formalin (negative control sample) remained non-infected after 2 passages.

### 3.4. Ethanol and Sodium Hypochlorite Disinfection of NiV

The effects of ethanol at 80%, 40%, 30% and 20% (*v*/*v*) and sodium hypochlorite at 10%, 1%, 0.5% and 0.1% (*v*/*v*) against NiV were tested for a contact time of 1 or 5 min. The samples were not mixed intentionally, but they did often coalesce to one large droplet of liquid. After the 1- or 5-min contact time, the samples were washed three times to remove the toxic components. 

**Figure 3:**
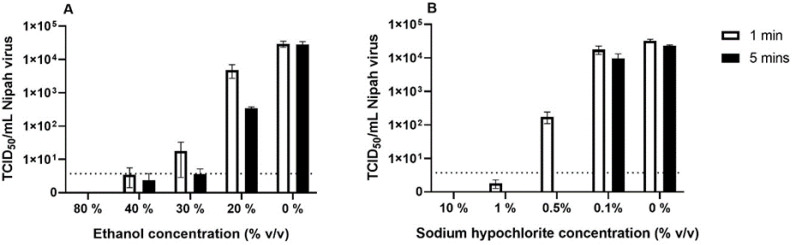
Efficacy of ethanol and sodium hypochlorite against NiV. Ethanol (**A**) or sodium hypochlorite (**B**) at a range of concentration percentages were tested for their effect on the titre of NiV with a 1 min (white bars) or 5 min (black bars) contact time. The titre values are means from multiple replicates ± SEM. Dotted line indicates the limit of detection of the TCID_50_ assay.

The samples were tested for a reduction in the titre of viable virus by a TCID_50_ assay (Figure 3), and also for the presence of low levels of virus by passage in cell culture. For the untreated control samples, TCM was added, and the samples were washed three times. The mean titre of NiV that was recovered was 3 × 10^5^ TCID_50_/mL.

For 1 min contact time, only 80% ethanol and 10% sodium hypochlorite (both *v*/*v*) were able to reduce the titre of NiV to un-detectable levels by TCID_50_ assay (Figure 3), and no viable virus was detected after passage in cell culture. Sodium hypochlorite at 1% (*v/v*) was also very effective against NiV; in two-thirds of the replicates that were tested, no viable virus was recovered after 1 min, and in the remaining third of the samples, the titre of the virus recovered was at the limit of detection of the assay. The two samples that resulted in a viral titre appeared in those flasks from the second passage; the remaining samples were negative throughout the passages. No NiV was detected by TCID_50_ assay or in the flasks when the contact time was increased to 5 min with 1% sodium hypochlorite (Figure 3A). When the sodium hypochlorite was at 0.5%, there was a reduction in viable virus with 1 min contact time, but still viable virus could be recovered from all of the samples that were tested. Increasing the contact time to 5 min reduced the NiV titre to below detectable limits in the flasks and by TCID_50_ assay. When the sodium hypochlorite was at 0.1%, it had a minimal effect on NiV titre, with 1- or 5-min contact time (Figure 3A).

Ethanol was less effective against NiV, with only a concentration of 80% resulting in no viable virus by TCID_50_ assay or after passage in cell culture. Ethanol at a concentration of 20% caused a minimal reduction in the titre of viable virus with 1 or 5 min contact time (Figure 3B). Contact with ethanol at a concentration of 30% or 40% resulted in a reduction in the titre of viable virus. At 30 and 40% ethanol, low levels of NiV (<20 TCID_50_/mL for 40% ethanol and <100 TCID_50_/mL for 30% ethanol) were still recovered from some replicates (Figure 3B). For 40% ethanol, two-thirds of the flasks remained un-infected throughout passage, and for 30% ethanol, half the flasks remained un-infected.

## 4. Discussion

In humans, NiV has been detected in nasal and oropharyngeal secretions as well as saliva, and the presence of the virus within these respiratory secretions means that aerosols or droplets could play a role in person-to-person transmission [14,15]. Susceptible laboratory animals, including hamsters and non-human primates have successfully been infected via the aerosol route, indicating that NiV may prove infectious via the inhalational route. However, the aerosol transmission of NiV between infected and non-infected hamsters has not been observed [16]. Additionally, there is insufficient data on aerosolized virus in health-care settings to fully understand the role of aerosols in the transmission of NiV. Human activities such as speaking and coughing produce particles in a range of sizes [17], and laboratory processes and medical procedures also generate aerosol particles which will range in size [5,6]. NiV may have pandemic or bioterrorism potential [3,4] and therefore, the stability of NiV that is contained within aerosols should be considered. 

In this study, NiV was shown to be relatively stable in an aerosol when it was under ambient conditions and in the dark. In recent years, we have studied the aerosol decay rate of a number of emerging viruses of concern [9,10,11]. Under comparable experimental conditions, the decay rate of NiV is similar to the mean values that were obtained for Ebola virus [11]. Although the comparison of aerosol data between different institutes can be subject to more variability due to different experimental parameters, our values for NiV are not that dissimilar to those that were obtained for Influenza virus under similar aerosol and environmental parameters [18]. The data that were obtained for the survival of NiV within a small particle aerosol in the dark, may be considered a baseline or ‘worse-case’ scenario for assessing the hazard or for modelling of aerosolised NiV. Larger aerosol particles will not persist for as long as they will drop out, and sunlight is known to inactivate viruses within aerosols, therefore, small particle aerosol in the dark is a condition that is most favourable to aerosol stability. The data on the stability of NiV in small particle aerosols can contribute to modelling (including transmission modelling) and advice on risk assessments and control measures. Aerosols arising from human activity are heterogeneous in their particle size, and hence, the data that are presented do not, on their own, directly inform the likely risk of transmission.

The evaluation of the aerostability of NiV within other matrices such as saliva, within larger particle aerosols, or under other more challenging environmental conditions (such as higher temperatures, more extreme values of relative humidity and exposure to simulated sunlight) which are known to have an impact on viral aerosol survival [19,20], may also help inform the risk that is posed by NiV. 

As of August 2022, there are no published data on NiV disinfection with sodium hypochlorite, and there is a single study that is focused on ethanol. (There are also no data on the other highly pathogenic members of the Henipahvirus family, Hendra virus). A 2019 review article reported that “NiV can be readily inactivated by soaps, detergents and commercially available disinfectants such as sodium hypochlorite” [21], but the paper that is cited does not have any efficacy data for Nipah virus, but it does suggest the use of 0.5% sodium hypochlorite for infection control [22]. We have looked at the efficacy of alcohol and bleach-based disinfection by testing a range of concentrations of sodium hypochlorite and ethanol. Only the highest concentrations that were tested (10% sodium hypochlorite and 80% ethanol) were fully effective and consistently resulted in no viable virus recovery after 1 min from a starting titre of just over 1 × 10^4^ TCID_50_ NiV. The use of ethanol, at all other concentrations that were tested, resulted in some level of viable virus being detected in at least one of the replicates, even when the contact time was increased to five minutes. For sodium hypochlorite, 5 min contact time with 1% and 0.5% was effective. Under those conditions, where no viable virus was recovered, a 4 log_10_ reduction in the viral titre was observed, which is considered the standard measure for disinfectant testing [23], although the testing was not performed to any quality standard.

The disinfection efficacy data that are produced in this study against NiV are comparable to the data that are published elsewhere for Ebola virus [24]. In those studies, 70% ethanol (the only concentration that was tested) was effective in reducing the viral titre to zero in 1 min for two of three variants that were tested (Mayinga and Kikwit), and within 2.5 min for the third variant (Makona) [24]. Sodium hypochlorite at 1% or 0.5% both required 5 min to reduce the titre of all three Ebola virus variants to zero [24]. Ethanol (at 70%) and “household bleach” at a 1:49 working concentration were also effective against SARS-CoV-2 after a 5-min contact time [25]. In a recently published study that was looking at quaternary ammonium compounds and ethanol against NiV, reduction in the viral titre was achieved with 38%, 57% and 76% ethanol, but at 19% ethanol, an 8-min contact time was needed to achieve a significant reduction in the viral titre [26]. 

In addition to sodium hypochlorite and ethanol, good disinfection efficacy has been achieved with a single concentration and a short contact time of several quaternary ammonium-based disinfectants against NiV (unpublished observations). Disinfection data provides useful fundamental knowledge for NiV disinfection that can be applied to laboratories that are performing research or in hospitals and clinical settings for people who are working with patients that are infected with NiV. As ever, with this type of work there are many more disinfectants, concentrations and contact times that could be investigated, and the work could also be extended to look at its efficacy in various matrices such as blood, where different parameters may be needed to achieve the same level of knockdown. 

The inactivation of NiV may be required for the exploitation of virus or virus-containing samples during research. Heat treatment at 56 °C or 60 °C has been shown to be effective at reducing the titre of NiV, as has UV irradiation for 30 min [27]. The reduction in the viral titre of NiV in palm sap has also been demonstrated at 70 °C and 100 °C [28]. The inactivation of NiV by formalin was recently demonstrated with the complete inactivation of infectious NiV after its fixation for 24  h in 10% Neutral Buffered Formalin, as shown in seven of the eight organ samples that were tested (one liver sample required 48 h incubation) [29]. In the present study, comparable results have been demonstrated using different methods and the testing 10% formalin against NiV in tissue culture medium for 24 h. This provides the proof of principle data to allow formalin-fixed samples to be removed safely from BSL-4 laboratories for their downstream exploitation at lower microbiological containment levels. Similar work will be carried out with nucleic acid extraction reagents to allow for the safe removal of RNA for potential future diagnostic and detection purposes. Neutral buffered formalin is one of 14 inactivation methods to be recently described [30]. NiV in lungs was inactivated by two rounds of 24-h incubation [30]. 

In conclusion, we report novel data on the survival of NiV in small particle aerosols, and on disinfection of NiV, which will help to inform risk assessments and control measures for those that are working with the virus in research or in outbreak situations. We have also added to data on the inactivation of NiV by neutral buffered formalin, which can be used to develop methods for exploiting samples from animals that are infected with NiV for histopathological analysis. This, in turn, will help in understanding disease pathogenesis and in the assessment of the efficacy of medical countermeasures in in vivo models.

## Data Availability

The data presented in this study are available in the article. © Crown copyright (2022), Dstl. This material is licensed under the terms of the Open Government Licence except where otherwise stated. To view this licence, visit http://www.nationalarchives.gov.uk/doc/open-government-licence/version/3 (accessed on 12 September 2022) or write to the Information Policy Team, The National Archives, Kew, London TW9 4DU, or email: psi@nationalarchives.gov.uk.
